# End-to-end non-invasive ECG signal generation from PPG signal: a self-supervised learning approach

**DOI:** 10.3389/fphys.2026.1694995

**Published:** 2026-02-05

**Authors:** Murat Yalcin, Marc Erich Latoschik

**Affiliations:** Human-Computer Interaction (HCI) Group, University of Würzburg, Würzburg, Germany

**Keywords:** deep learning, electrocardiogram, generative adversarial network, healthcare, photoplethysmogram, physiological signals, self-supervised learning, signal processing

## Abstract

Electrocardiogram (ECG) signals are frequently utilized for detecting important cardiac events, such as variations in ECG intervals, as well as for monitoring essential physiological metrics, including heart rate (HR) and heart rate variability (HRV). However, the accurate measurement of ECG traditionally requires a clinical environment, thereby limiting its feasibility for continuous, everyday monitoring. In contrast, Photoplethysmography (PPG) offers a non-invasive, cost-effective optical method for capturing cardiac data in daily settings and is increasingly utilized in various clinical and commercial wearable devices. However, PPG measurements are significantly less detailed than those of ECG. In this study, we propose a novel approach to synthesize ECG signals from PPG signals, facilitating the generation of robust ECG waveforms using a simple, unobtrusive wearable setup. Our approach utilizes a Transformer-based Generative Adversarial Network model, designed to accurately capture ECG signal patterns and enhance generalization capabilities. Additionally, we incorporate self-supervised learning techniques to enable the model to learn diverse ECG patterns through specific tasks. Model performance is evaluated using various metrics, including heart rate calculation and root mean squared error (RMSE) on two different datasets. The comprehensive performance analysis demonstrates that our model exhibits superior efficacy in generating accurate ECG signals (with reducing 83.9% and 72.4% of the heart rate calculation error on MIMIC III and Who is Alyx? datasets, respectively), suggesting its potential application in the healthcare domain to enhance heart rate prediction and overall cardiac monitoring. As an empirical proof of concept, we also present an Atrial Fibrillation (AF) detection task, showcasing the practical utility of the generated ECG signals for cardiac diagnostic applications. To encourage replicability and reuse in future ECG generation studies, we have made both the dataset and the code publicly available.

## 1 Introduction

Cardiovascular diseases (CVDs) represent the leading cause of mortality worldwide, accounting for 32% of all global deaths^1^ (WHO, 2019). In 2021, out of 20.5 million deaths attributed to CVDs, approximately 80% occurred in low- and middle-income countries (Cesare et al., 2024). A primary factor contributing to this high mortality rate is the inadequate provision of primary healthcare and the limited availability of accessible, on-demand health monitoring systems. The electrocardiogram (ECG) is recognized as a critical tool for continuous health monitoring and is vital for identifying individuals at elevated risk of future cardiovascular events or mortality. Regular ECG monitoring has demonstrated effectiveness in the early detection of CVDs (Rosiek and Leksowski, 2016).

The ECG measures the heart’s electrical activity and provides essential insights into cardiovascular health. While the 12-lead ECG is considered the clinical gold standard, even simpler alternatives, such as Holter ECG, are often cumbersome and impractical for continuous monitoring. The process of attaching multiple electrodes can cause discomfort, and signal quality may degrade over time due to variations in skin-electrode impedance. Although significant research has focused on developing wearable devices that facilitate continuous ECG monitoring suitable for daily use, these efforts have largely been unsuccessful.

Photoplethysmogram (PPG), an optical technique used to detect volumetric changes in blood within peripheral circulation, is commonly integrated into wearable devices like smartwatches. PPG holds potential for generating ECG-like representations and provides valuable cardiovascular insights. With advancements in wearable and mobile devices, such as smartwatches and smartphones, PPG has become the industry standard for continuous heart rate (HR) monitoring, valued for its simplicity, user-friendliness, and cost-effectiveness (Park et al., 2022; Castaneda et al., 2018). However, PPG has several limitations, including inaccuracies in HR estimation and susceptibility to external factors such as skin tone, skin type variability, motion artifacts, and signal interference (Bent et al., 2020). Motion artifacts, in particular, can significantly distort PPG signals, making it challenging to capture precise cardiovascular information. Constructing a dataset that includes PPG/ECG data with motion artifacts could be beneficial for developing more robust algorithms. In this context, Virtual Reality (VR) games offer a promising alternative for simulating such conditions and addressing this challenge (Halbig and Latoschik, 2021).

Since PPG does not measure the electrical activity of the heart but rather the mechanical response (blood volume changes) to the heart’s pumping action, it cannot capture detailed electrical events except the systolic peak (due to arterial blood volume increase) and the dicrotic notch (related to the closure of the aortic valve). Compared to PPG, the ECG waveform encompasses critical details about cardiac activity; for instance, the P wave represents atrial depolarization, the R wave representing ventricular depolarization (Feher, 2012), and a prolonged PR interval may indicate a delay in conduction through the atrioventricular node, characteristic of a first-degree heart block, which points to potential dysfunction in the heart’s electrical conduction system (Mammen et al., 2004).

Given these considerations, a significant gap exists between the demand for continuous wearable ECG monitoring and the detailed information it delivers, and the available non-invasive, mobile, and cost-effective solutions. While PPG lacks the distinct waves of the ECG, its waveform still contains periodic components and cardiac cycle. This inherent relationship between PPG and ECG signals does allow for PPG-to-ECG translation using suitable methods, particularly leveraging advanced machine learning and signal processing techniques Banerjee et al. (2014); Zhu et al. (2021). The widespread adoption of wearable devices that continuously collect PPG signals has resulted in the availability of large-scale data, motivating the utilization of the cardiovascular relationship between PPG and ECG to generate ECG waveforms from PPG measurements using deep learning (DL) models (Tang et al., 2022). This approach has the potential to enable low-cost ECG screening for continuous and long-term monitoring, merging the comprehensive clinical insights provided by ECG signals with the accessibility of PPG data. To this end, we propose an end-to-end Transformer-based Generative Adversarial Network (GAN) model to generate ECG signals from PPG inputs.

Additionally, enhancing the representation learning process and improving knowledge retention is essential for effective PPG-to-ECG translation. By integrating self-supervised learning, our model learns robust and transferable representations of ECG signals through auxiliary tasks such as contrastive learning and masked signal modeling. Self-supervised learning also mitigates catastrophic forgetting—a common and critical issue in GAN training where the model abruptly loses previously acquired knowledge when learning new information (Thanh-Tung and Tran, 2020). To address this, we design auxiliary tasks specifically for the discriminator to help capture the intrinsic structure of ECG signals, thereby enhancing the model’s ability to generalize across different datasets and tasks. This ensures robust feature retention and transfer, ultimately improving the quality and reliability of the generated ECG signals (Chen et al., 2019).

In this study, our contributions are summarized as follows:

- We collected PPG and ECG data using consumer-grade wearable sensors while participants engaged in a VR game. To simulate real-life conditions as closely as possible, data collection was conducted in an unstructured environment, allowing for unrestricted movement and realistic actions. The resulting dataset, named Who is Alyx? (Rack et al., 2023), has been made publicly available.
- We introduced a novel Transformer-based GAN model to accurately synthesize ECG waveforms from PPG signals. For the first time, we implemented ECG generation on the Who is Alyx?. Our model demonstrated superior performance on both the MIMIC III benchmark dataset and the Who is Alyx? dataset, compared to state-of-the-art methods in the literature, as evaluated by various metrics. Additionally, we explored the impact of different signal lengths on the quality of ECG synthesis and examined distributional similarities between real and generated ECG signals.
- We were the first to leverage a self-supervised framework for ECG signal generation through multi-task ECG representation learning. In this context, we utilized well-known three different paired PPG-ECG datasets for pre-training to investigate the effect of the self-supervised approach on ECG synthesis.
- To evaluate the generalization capability of the proposed model, we employed a leave-one-subject-out (LOSO) cross-validation strategy. This approach enabled us to assess the model’s performance on previously unseen participants, thereby addressing to prevent potential validation concerns. Furthermore, to promote replicability and facilitate future research in the field of ECG generation, we have shared original dataset (Who is Alyx? (Rack et al., 2023)) and have made our code publicly available.^2^
- To demonstrate the practical utility of the generated ECG data, we conducted an Atrial Fibrillation (AF) detection study using two deep learning classifiers on a dedicated dataset. We provided a detailed analysis of the contribution of generated ECG data to the classification task, including a comprehensive evaluation of baseline variability in both real and generated ECG data.

The remainder of this paper is organized as follows. Section 2 reviews the existing literature on PPG-to-ECG generation methods. The experimental design and datasets are described in Section 3. Section 4 introduces the proposed Transformer-based GAN architecture. The data preprocessing pipeline, self-supervised pre-training strategy, fine-tuning procedure, hyperparameter optimization, and overall implementation details for the PPG-to-ECG generation framework are presented in Section 5. The evaluation metrics are defined in Section 6, while quantitative and qualitative results are reported in Section 7. A practical application of the proposed model for AF detection is presented in Section 8. Finally, Sections 9, 10 provide a discussion of the findings and concluding remarks, respectively.

## 2 Related work

Previous studies have explored the relationship between PPG and ECG signals, highlighting that certain characteristics of heartbeats, including key parameters of an ECG such as heart rate, heart rate variability, etc., are also reflected in PPG signals (Weinschenk et al., 2016; Banerjee et al., 2014), though not with the same precision as direct ECG measurements.

Research on ECG generation is relatively limited. Some early studies have been discussed the concept of generating ECG signals with a strong focus on understanding and modeling ECG waveform morphologies (Sayadi et al., 2010; McSharry et al., 2003). Typically, statistical modeling is employed to generate synthetic ECG signals on a beat-by-beat basis, often using RR intervals, where individual beats are sequentially assembled based on specific beat information (Maheshwari et al., 2014; Craven et al., 2017).

Recent approaches for reconstructing ECG signals from PPG have explored various signal processing techniques. (Zhu et al., 2021). proposed a method utilizing discrete cosine transform (DCT), where PPG onsets were aligned with ECG R-peaks, followed by de-trending, cycle segmentation, and linear interpolation to standardize segment lengths. A linear regression model was then trained to map DCT coefficients of PPG to those of ECG. Despite its structured design, this approach suffered from limited generalizability to unseen subjects, inadequately modeled the inherent non-linearities between PPG and ECG, and lacked comparative evaluation in terms of heart rate estimation accuracy. Alternatively, (Tian et al., 2023), introduced a cross-domain joint dictionary learning framework, employing a correlation matrix to translate PPG to ECG. While promising, their method exhibited poor performance in subject-independent settings and was particularly vulnerable to motion artifacts in distorted PPG recordings. More recently, (Shome et al., 2024), proposed a Region-Disentangled Diffusion Model for reconstructing ECG signals from PPG, highlighting diffusion-based generative modeling as a potential direction for improving translation fidelity. Moreover, such approaches typically depend on extensive pre-processing and handcrafted features, potentially introducing biases and limiting adaptability. For instance, (Belhasin et al., 2025), introduced artificial noise injection on clinically acquired datasets to mimic and mitigate motion artifacts.

Several studies in the literature have employed machine learning-based methods to address ECG generation tasks, with recent trends favoring deep learning-based methods. For instance, (Banerjee et al., 2014), deployed Support Vector Machine (SVM) that trained multiple classifiers using features extracted from the time and frequency domains to estimate ECG intervals (PR, QRS, QT, and RR intervals) from selected features of PPG.

More recent contributions including (Tang et al., 2022; Abdelgaber et al., 2023; Guo et al., 2024) proposed bidirectional LSTM-based models for generating ECG waveforms from PPG, often requiring R-peak detection or beat-based segmentation. (Tang et al., 2022).’s generated ECG signal windows of varying lengths, which were then stitched together to form the final ECG segments. Although capable of constructing long ECG signals, this method compromised performance measures and used a dataset collected in a clinical setting without motion artifacts, raising concerns about its applicability to real-world scenarios involving ECG reconstruction. Additionally, (Vo et al., 2024), introduced an attention-based deep state-space model for PPG-to-ECG generation, demonstrating its utility through downstream AF detection. Building upon the insights from prior studies, there is a need for an end-to-end deep learning approach that captures the non-linear relationship between PPG and ECG signals without relying on manual feature engineering. Training on datasets with real-world motion and artifacts is essential to reflect practical conditions. Additionally, the model should be evaluated on unseen subjects using metrics like heart rate estimation to assess the fidelity of the generated ECG.

### 2.1 Generating ECG using GAN models

GANs (Goodfellow et al., 2014) have demonstrated significant potential in the medical domain, including applications such as medical image synthesis, noise reduction, tumor detection, and lesion segmentation (Lan et al., 2020), highlighting the growing importance of GANs in medical data analysis. There have been studies using GAN models for bio-signal data augmentation including ECG signals in the medical and healthcare domains (Zhou et al., 2021; Yalcin et al., 2024; Delaney et al., 2019). Specifically, in the realm of ECG data augmentation, GANs are employed to generate realistic synthetic ECG signals, thereby mitigating the challenge of limited data availability and enhancing the training of machine learning models for tasks like arrhythmia detection and other cardiac-related diagnostics (Rahman et al., 2023; Berger et al., 2023). For example, (Zhu et al., 2019), described a synthetic ECG signal generation model using a bidirectional LSTM-CNN-based GAN architecture that generated ECG from Gaussian noise which is input for the generator, achieving 0.257 mV RMSE and 0.728 Fréchet Distance (FD) values. (Golany et al., 2020). used a deep convolutional GAN (DCGAN) model to synthesize ECG signals, aiming to enhance ECG heartbeat classification performance.

With specific case of inputting the GAN model with PPG to generate ECG is very limited. (Sarkar and Etemad, 2020). deployed an attentive CycleGAN architecture with a dual discriminator to synthesize ECG signals from PPG, achieving an RMSE of 0.364 mV. However, their model struggled with low-quality outputs for highly noisy PPG signals. Also, their GAN model was encountered the challenge of overcoming unstable training resulted in occasional but critical random oscillations. (Vo et al., 2021). utilized a Wasserstein GAN with PPG inputs for ECG synthesis on MIMIC II dataset (Saeed et al., 2002). They did not validate their model with noisy PPG signals where the source of noise is from real-life activities. We discuss similar works in Section 9, with a detailed comparison presented in Table 8.

The Transformer architecture, characterized by its exclusive use of self-attention mechanisms, has revolutionized machine learning by eliminating the need for recurrent layers (Vaswani et al., 2017). Its success across diverse domains such as emotion recognition (Yalcin and Latoschik, 2024), language translation, etc., highlights its potential applicability to time-series signal processing tasks. In the areas of arrhythmia and anomaly detection, several studies have achieved state-of-the-art results employing both supervised and unsupervised methods with various Transformer-based models (Hu et al., 2022; Shah et al., 2024; Alamr and Artoli, 2023).

Notably, (Lan, 2023), explored a Transformer-only architecture (without GAN) for patch-based PPG-to-ECG translation. However, their evaluation was confined to clinically acquired datasets, leaving the model’s robustness under real-world, motion-intensive conditions unexamined. Moreover, the patch-based design requires specialized architectural choices and numerous hyperparameters, increasing the overall complexity of the generation pipeline. Despite some successful studies has shown great result with GAN and transformer combination, no prior study has attempted to integrate a Transformer model within a GAN framework for PPG-to-ECG signal generation and makes that task unexplored. Consequently, the impact of the Transformer’s generalization capability on this specific task has yet to be thoroughly investigated.

### 2.2 Self-supervised learning approaches

Recent advancements in machine learning and deep learning have highlighted the effectiveness of self-supervised models in acquiring generalized and robust representations. Self-supervised learning is a machine learning approach in which models are trained using automatically generated pseudo-labels rather than manually annotated ones. This approach has been successfully applied across a wide range of fields, including computer vision (Chen et al., 2019; Kocabas et al., 2019; Wang et al., 2019), speech processing (Tagliasacchi et al., 2019), and natural language processing (Wu and Weld, 2010). As implementation on ECG signals, two noticeable studies can be shown. For the first one, (Vazquez-Rodriguez et al., 2022), utilized self-supervised learning to address the challenges posed by the limited size of emotionally labeled datasets in classification tasks. Secondly and similarly, (Sarkar and Etemad, 2022), applied self-supervised learning to ECG signals for emotion recognition, demonstrating a significant performance improvement compared to fully-supervised training.

In the context of GAN models, there are limited studies incorporating self-supervised learning. For example, (Chen et al., 2019), introduced a GAN model that integrates adversarial and self-supervised learning for natural image synthesis, aiming to bridge the gap between unconditional and conditional models. This approach mitigated the problem of catastrophic forgetting, resulting in stable training and optimized representations. Additionally, it was shown that a small amount of data could be used to fine-tune the model after self-supervised learning.

## 3 Conceptual overview

Despite aforementioned advancements, the application of self-supervised learning specifically for ECG signal generation has not been extensively explored, particularly in the context of PPG-ECG paired generation. This paper addresses this gap by developing a robust Generative Adversarial Network (GAN) model to generate ECG signals from PPG inputs, supported by self-supervised learning techniques. Our goal is to achieve high-performance metrics, especially on datasets characterized by high noise levels and motion artifacts. To this end, we propose a Transformer-based GAN model designed to overcome these challenges and improve the reliability and accuracy of ECG synthesis from PPG data. VR technology has shown to be able to evoke a large variety of interesting and important psychological and physiological responses (Halbig and Latoschik, 2021), including stress, anxiety, and fear (Yalcin and Latoschik, 2024). Accordingly, it is now an accepted alternative method applied in psycho therapy, e.g., in the treatment of specific anxiety disorders or PTSS, etc. (Andersen et al., 2023). VR environments can be used to create high levels of immersion, i.e., sensorimotor contingencies comparable to experiences in the real physical world, including a rich variety of full body motions and interaction. Hence, VR provides excellent possibilities to evoke and measure physiological data even with lab-bound devices while allowing quite large degrees of freedom. This immersive engagement results in more diverse and dynamic ECG waveforms, reflecting the emotional and physical state of the user (Halbig and Latoschik, 2021).

The combination of emotional (stress, fear, anxiety, etc.) and physical stimuli in VR gaming offers a unique opportunity to explore ECG generation solutions that account for these multifaceted effects. Unlike sedentary activities, VR gaming often involves frequent physical movements, such as turning, crouching, and reaching. These movements introduce additional variability, including motion artifacts in both ECG and PPG signals, which are often measured using wearable devices. These movement-induced artifacts pose significant challenges for ECG generation, as they can distort the signal quality, making it more difficult to process.

### 3.1 VR game and measurement design

For this study, we selected “Half Life-Alyx” (Valve Corporation, 2020), a VR prequel to the renowned series by Valve Corporation. Although not explicitly a horror game, it integrates unsettling elements, particularly in a VR context. The game’s detailed graphics, meticulously designed environments, suspenseful pacing, and encounters with terrifying creatures such as head-crabs contribute to a pervasive sense of fear. The sophisticated sound design, incorporating ambient noises and environmental hazards, further heightens the player’s sense of vulnerability, stress, and fear.

**FIGURE 1:**
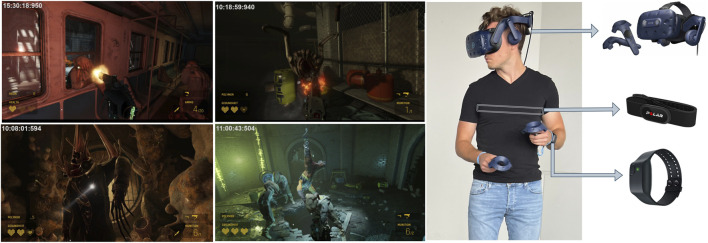
First two columns shows the screenshots of the VR game (Half Life-Alyx) that participants played during data collection. Last column shows an equipped participant and the respective sensors used during the experiments in detail.

To capture the participants’ physiological signals during their VR game experience, we employed three different devices. Recognizing the advantages of wearable sensors in terms of cost, ease of use, and portability, we selected the Polar H10 (Polar Electro Oy, Finland), an electrode-based chest strap, and the Empatica E4 (Empatica Inc., United States), a medical-grade wristband. Both devices transmit data to a computer via Bluetooth communication. Additionally, we used the HTC Vive Pro as the headset (HMD) to collect eye-tracking data during the gameplay. These sensors are straightforward to deploy and can be utilized in various scenarios with minimal setup effort. Screenshots of the Half Life-Alyx game and images of a participant equipped with the sensory devices are shown in Figure 1.

### 3.2 Ethical consideration

The study was approved by the Research Ethics Committee of the Institute for Human-Computer Media (MCM) of human sciences of the University of Würzburg at 30th May of 2022 and was conducted in accordance with the local legislation and institutional requirements. The participants were recruited through the participant recruitment system of our faculty and gave their full consent to publish and process the collected and anonymized data. Every participant was fully informed about the intents and purposes and the procedure of the data acquisition.

### 3.3 Data collection

The study involved 34 participants (14 female, 20 male), aged between 21 and 33 years, with a mean age of 25.3 years. Only two participants had prior experience playing the selected game. Participants were equipped with the sensory devices, and connections were established between the sensors and the measurement engine (Yalcin et al., 2022), a custom software developed as part of the VIA-VR project (von Mammen et al., 2019), primarily using Python. Data streaming was initiated simultaneously for all sensors. Initially, we collected 3 min of baseline data while participants freely moved around with the sensors and selected the desired game chapter from the game menu. Throughout the study, an instructor was present to monitor participants in both the real and virtual environments, ensuring uninterrupted gameplay. All participants began their first session in “Chapter 1″ of the game and continued playing without further instructions. Each session lasted approximately 45 min, regardless of the participant’s progress within the game. The procedure was repeated for a second session on a different day, with participants starting from “Chapter 3.”

We collected ECG data from the Polar H10 at a sampling rate of 130 Hz and PPG data from the Empatica at 64 Hz. Although the dataset also includes other physiological and movement-related data, such as Acceleration (ACC), Electrodermal Activity (EDA), and Peripheral Body Temperature (TEMP), as detailed in the “Who is Alyx?” study (Rack et al., 2023), this study specifically concentrated on utilizing the ECG and PPG data for further analysis. The “Who is Alyx?” dataset is publicly available via GitHub repository: https://github.com/cschell/who-is-alyx. The dataset is intended for research purposes only.

### 3.4 MIMIC III and MIMIC PERform AF datasets

In addition to our Who is Alyx? dataset, we employed the MIMIC III matched waveform database (Johnson et al., 2016; Moody et al., 2020) to evaluate the generalization capability and benchmark performance of our model on a widely recognized dataset. MIMIC III contains multiple physiological signals recorded from intensive care unit (ICU) patients and serves as an extended and enhanced version of the earlier MIMIC II database (Saeed et al., 2002). Each record is 5 min long, with simultaneous ECG and PPG signals sampled at 125 Hz. For this study, we randomly selected 100 records from different subjects using lead II ECG and PPG signals, aligning with the sample size commonly used in related works (Zhu et al., 2021; Tang et al., 2022).

For the Atrial Fibrillation (AF) detection analysis, we additionally utilized the publicly available annotated MIMIC PERform AF dataset (Charlton et al., 2022; Bashar et al., 2019). This dataset consists of 20-min waveform recordings from 35 ICU patients, comprising 19 patients with AF and 16 patients in normal sinus rhythm (non-AF). The dataset is a curated subset of the MIMIC III matched waveform database and provides high-quality annotations suitable for evaluating arrhythmia detection models.

## 4 Transformer-based GAN model

Since our ECG generation model is based on the GAN architecture, we begin with a brief overview of its functioning. A Generative Adversarial Network (GAN) (Goodfellow et al., 2014) comprises two components: a generator (G) and a discriminator (D) . The generator synthesizes data, while the discriminator distinguishes between real and generated samples. In our context, the generator learns to map PPG signals to ECG data. Its goal is to deceive the discriminator into classifying synthetic data as real. Through iterative training, guided by a loss function that captures the discriminator’s accuracy, the generator improves, and its output distribution gradually aligns with that of real data.

The generator $G_{\theta_{g}}$ operates as a directed latent variable z model that deterministically generates samples x from the latent space $\left(z∼p_{z}\right)$ with optimization (minimize) of $\theta_{g}$ , generator parameters. Given that the discriminator $D_{\theta_{d}}$ aims to classify samples as real (x) or fake with optimization (maximize) of $\theta_{d}$ , discriminator parameters, the adversarial game between the generator (G) and the discriminator (D) can be formalized through an objective function, V(D,G) , which frames the interaction as a classification problem. Here, the expected values of the variables drawn from distributions are denoted as E , real x samples drawn from real data distribution denoted as $\left(x∼p_{\mathrm{data}}\right)$ and latent z samples drawn from latent noise distribution, often a Gaussian distribution denoted as $\left(z∼p_{z}\right)$ . The general form of the objective function (Goodfellow et al., 2014) is expressed in Equation 1:

$$\min_{\theta_{g}}\max_{\theta_{d}}V\left(D,G\right)=\left[E_{x∼p_{\mathrm{data}}}\log D_{\theta_{d}}\left(x\right)+E_{z∼p_{z}}\log\left(1-D_{\theta_{d}}\left(G_{\theta_{g}}\left(z\right)\right)\right)\right]$$ (1)

During the training process, GANs often encounter a prevalent issue known as mode collapse, wherein the generator network persistently produces identical outputs. This limitation significantly reduces the diversity of the generated data and diminishes the generator’s ability to accurately capture the complex distribution of real-world data. In the following section, we introduce our approach to addressing this problem.

### 4.1 Wasserstein loss with gradient penalty

To address the mode collapse problem, we employed the Wasserstein GAN (WGAN) loss function (Arjovsky et al., 2017), which minimizes the Wasserstein (Earth Mover’s) distance between real and generated data distributions. This approach offers improved gradient flow and robustness to hyperparameter variations compared to traditional GAN loss.

In our approach, the discriminator, also referred to as the “critic” in WGAN models, D , is trained to differentiate between real and synthetic ECG signals, while the generator neural network G is trained to generate ECG signals from PPG signals, with the objective of making the generated ECGs indistinguishable by the discriminator. Let’s denote individual segments (time windows) as p and the corresponding ground-truth ECG segments as e . For the mapping function $G_{E}:P\to E$ , and discriminators $D_{E}$ , with ECG signals e drawn from the data generating distribution $ECG_{\mathrm{data}}\left(e\right)$ and signals p drawn from the input prior $PPG_{\mathrm{data}}\left(p\right)$ , the generator G and the discriminator D jointly optimize the following non-artificial $L_{G}$ (generator) and $L_{D}$ (discriminator) loss functions, as formulated in Equations 2, 3 respectively:

$$L_{G}=-E_{p∼P_{\text{PPG}}}\left[D\left(G\left(p\right)\right)\right]$$ (2)

$$L_{D}=-E_{e∼P_{\text{ECG}}}\left[D\left(e\right)\right]+E_{p∼P_{\text{PPG}}}\left[D\left(G\left(p\right)\right)\right]$$ (3)

To maintain the 1-Lipschitz continuity constraint required for WGANs (Gulrajani et al., 2017), which is essential for the proper functioning of the discriminator (Arjovsky et al., 2017), a gradient penalty is applied between the real and synthetic data distributions (Gulrajani et al., 2017). We followed this strategy and included this penalty term to ensure that the gradients of the discriminator with respect to its inputs do not exceed a norm of 1, thereby promoting stable training. The gradient penalty term, denoted as $L_{\text{GP}}$ , is defined in Equation 4. Here, the gradient operator is denoted as ∇ .

$$L_{\text{GP}}=\lambda E_{\hat{x}∼P_{\hat{x}}}\left[{\left(\|\nabla_{\hat{x}}D\left(\hat{x}\right){\|}_{2}-1\right)}^{2}\right]$$ (4)

Here, $\hat{x}$ is an interpolated sample between real and generated data points, computed in Equation 5:

$$\hat{x}=\epsilon e+\left(1-\epsilon \right)G\left(p\right)$$ (5)

where ϵ is a random number sampled from the uniform distribution U(0,1) , and λ is the scaled factor of gradient penalty coefficient. The effect of the gradient penalty coefficient was analyzed during hyperparameter optimization (see Section 5.4.1; Figure 5). Incorporating the gradient penalty, the complete loss for the discriminator can be written in Equation 6:

$$L_{D}=-E_{e∼P_{\text{ECG}}}\left[D\left(e\right)\right]+E_{p∼P_{\text{PPG}}}\left[D\left(G\left(p\right)\right)\right]+\lambda E_{\hat{x}∼P_{\hat{x}}}\left[{\left(\|\nabla_{\hat{x}}D\left(\hat{x}\right){\|}_{2}-1\right)}^{2}\right]$$ (6)

Finally, our adversarial Wasserstein objective function $L_{W_{\mathrm{GAN}}}\left(D,G\right)$ for the mapping $G_{E}:P\to E$ is Equation 7:

$$\underset{G\left(p\right)}{\min}\underset{D\left(e\right)}{\max}L_{W_{\mathrm{GAN}}}\left(L_{D},L_{G}\right)$$ (7)

### 4.2 ECG generator architecture

Generating ECG signals from PPG poses challenges in modeling both local waveform morphology and long-range temporal dependencies. To address these, we explored various GAN architectures. Traditional models like DCGAN (Radford et al., 2016), relying solely on convolutional layers, fail to capture sequence-wide dependencies, while SeqGAN (Yu et al., 2016), though tailored for sequence generation, is computationally intensive and unsuitable for high-resolution ECG synthesis.

In contrast, Transformer models have shown strong performance in both classification (Vazquez-Rodriguez et al., 2022) and generative tasks (Gong et al., 2022), owing to their self-attention mechanism, which effectively captures both local and global dependencies. Given these strengths, we adopted a hybrid architecture combining convolutional layers for local temporal feature extraction with a Transformer module to model sequential dependencies, enabling the generation of high-fidelity ECG signals.

**FIGURE 2:**
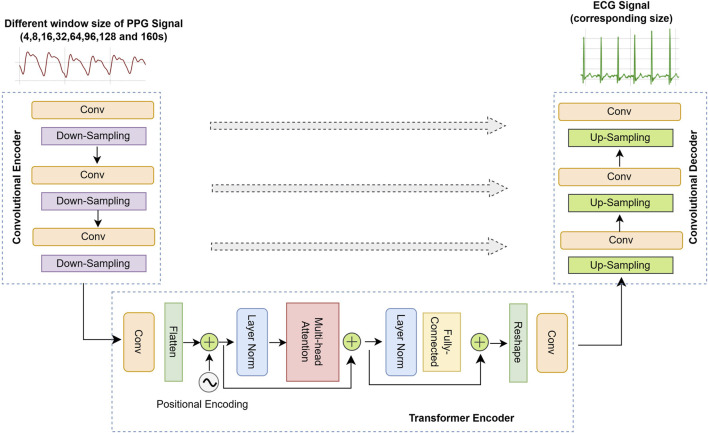
The overall ECG generator architecture model which consist of three branches: 1- The convolutional encoder (left-side) which receives different time length of PPG signal as input extracts spatial features. 2- The Transformer encoder is concatenated between convolution encoder-decoder with causal self-attention for long-range dependency modeling (bottom-side). 3- The convolutional decoder (right-side) up-samples feature maps and outputs the ECG signal with corresponding length. The grey arrows (middle-side) shows skip connections between the corresponding layers of the convolutional encoder-decoder. The feature maps from the encoder are concatenated with the upsampled feature maps in the decoder. This allows the network to combine high-resolution features from early layers with the high-level abstract features learned in deeper layers.

For the generator architecture of our GAN model, we integrated a UNet (Ronneberger et al., 2015) with a custom-built from-scratch Transformer model (Vaswani et al., 2017), utilizing this combination as the backbone generator of our GAN framework. The overall architecture of the ECG generator, comprising three primary branches, is illustrated in Figure 2.

#### 4.2.1 Convolutional encoder

The generator adopts a UNet-inspired encoder–decoder architecture based on 1-D convolutions. Convolutional networks offer superior parallelization and faster computation compared to recurrent models, while achieving comparable or better performance (Elbayad et al., 2018). The input consists of PPG segments ranging from 4 to 160 s (e.g., 520 samples for 4 s), with the output being ECG segments of matching length. The architecture compresses the input via downsampling to a bottleneck layer and reconstructs it through upsampling using transposed convolutions.

#### 4.2.2 Transformer encoder

Following the CNN encoder, a Transformer encoder was employed to further capture feature information using causal self-attention, which is crucial for modeling long-range dependencies. Initially, the feature map channels were expanded from 8 to 16 via a convolution layer. Given that the Transformer processes information in a token-to-token manner, the two-dimensional feature maps with a PPG segment were flattened into a sequence of tokens. A learned positional embedding (Vaswani et al., 2017; Karimi et al., 2021) was then added to the sequence. This step is crucial because, without positional information, the Transformer’s attention mechanism would be insensitive to sequence order due to its inherent arrangement invariance. After incorporating the positional encoding then fed into the Transformer encoder.

The Transformer encoder consists of alternating layers of multi-head self-attention blocks and multi-layer perceptron (MLP) blocks. Layer normalization (LN) (Ba et al., 2016) is applied before each block, and residual connections are added after each block. The MLP block consists of a two-layer fully connected feed-forward network incorporating Dropout (Srivastava et al., 2014) and the Gaussian Error Linear Unit (GELU) activation function (Hendrycks and Gimpel, 2016). After processing through the Transformer encoder, the feature maps are reshaped and compressed to align with the input dimensions of the convolutional decoder.

#### 4.2.3 Convolutional decoder

The convolutional decoder employs transposed convolutions, also known as fractionally-strided convolutions, to progressively increase the sequence length until the final layer, which utilizes the Tanh activation function. Both the encoder and decoder consist of L=4 layers. To ensure the preservation of information across down-sampled layers, skip connections are employed to link the output of layer i in the encoder with the output of layer L=i in the decoder.

### 4.3 Transformer discriminator

**FIGURE 3:**
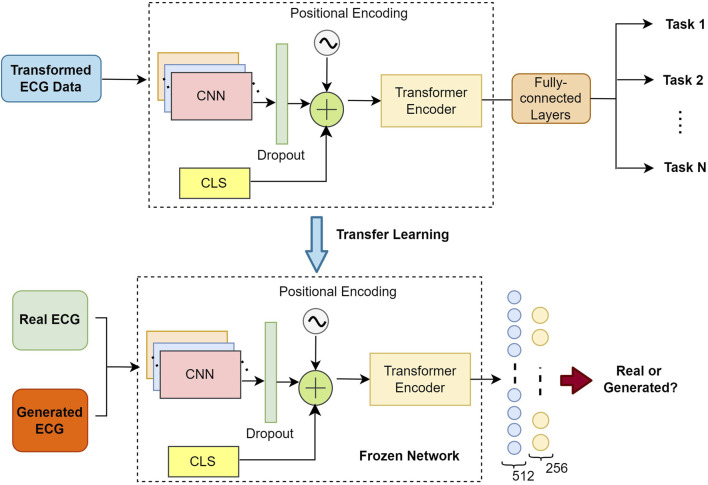
The overall scheme illustrates the implementation of self-supervised learning with the discriminator (shown in a dashed rectangle) and its subsequent use as a frozen network for fine-tuning our GAN model: 1- Top-side depicts the general steps of the self-supervised learning process (downstream task) using the discriminator. Transformed signals, generated from three datasets, are input into the discriminator to train it across six different tasks. 2- Down-side illustrates the partially frozen discriminator architecture, which is employed during the fine-tuning stage to distinguish between real and generated ECG signals.

The discriminator of our GAN model consist of Transformer architecture. The general architecture of the discriminator is illustrated with dashed rectangles in Figure 3.

To process ECG data using our Transformer discriminator model, the data were encoded into s feature vectors, where each vector represents a data sample with d dimensions. This encoding yielded a set of features $F=\left\{f_{1},\ldots ,f_{s}\right\}$ , where $f_{i}\in R^{d}$ . Adopting the BERT methodology (Devlin et al., 2019), the output of the Transformer includes an embedding of the classification token $\left(e_{\text{CLS}}\right)$ , alongside other signal representations. Through the attention mechanisms of the Transformer, $e_{\text{CLS}}$ aggregates information from the entire input signal as well as its contextualized representations. To incorporate the actual sequence order, positional information is added to each input fed into the Transformer. Specifically, the positional embeddings are summed with the features $F^{\prime }$ to form $Z=\left\{\text{CLS}+pe_{0},f_{1}+pe_{1},\ldots ,f_{s}+pe_{s}\right\}$ , where $pe_{i}\in R^{d}$ denotes the positional embedding for time-step i . After normalizing Z (Ba et al., 2016), the Transformer encoder generates contextualized representations E using h attention heads and l layers, formulated as ${\text{Transformer}}_{h\times l}\left(Z\right)=C=\left\{e_{\text{CLS}},e_{1},\ldots ,e_{s}\right\}$ . These representations C are then used for classifying the ECG data as real or synthetic.

The input encoder is composed of three layers of 1D Convolutional Neural Networks (CNN) with ReLU activation functions (Xu et al., 2015). Layer normalization (Ba et al., 2016) is applied to the first layer and at the encoder’s output. The kernel sizes for the layers are set to (65, 33, 17), with corresponding channel numbers of (64, 128, 256), and a stride of 1 for all layers. The Transformer’s signal encoder is empirically configured with a model dimension $d_{\text{model}}=256$ , 2 layers, and 2 attention heads, and a Fully Connected Network (FCN) size of $d_{\text{model}}\times 2=512$ . The FCN employed for predicting masked values consists of a single linear layer of size $d_{\text{model}}/2=128$ , followed by a ReLU activation function. An additional linear layer projects the output vector to a single value, corresponding to the predicted value of a masked point.

## 5 Learning contextualized representation

Motivated by the critical challenge of catastrophic forgetting in discriminators (Thanh-Tung and Tran, 2020), our objective is to enhance the discriminator’s ability to learn meaningful representations independently of the generator’s current performance. To address this issue, we employed recent advancements in self-supervised learning techniques for representation learning (Sarkar and Etemad, 2022). To further extend the generalization capabilities of our models, we utilized self-supervised learning by pre-training on multiple unlabeled ECG datasets. These pretext tasks were designed to learn robust feature representations, which were subsequently fine-tuned for the ECG signal generation process.

### 5.1 Self-supervised ECG representation learning

Although various signal transformations have been applied across different types of data (Saeed et al., 2019), for ECG data, we adopted the signal transformations proposed by (Sarkar and Etemad, 2022) and were the first to implement these transformations in our study for the purpose of ECG generation with GAN model. As part of our pretext tasks, we implemented six distinct signal transformation recognition tasks, as outlined below:

#### 5.1.1 Noise addition

Gaussian noise N(t) with zero mean and standard deviation $\sqrt{E_{N_{\text{avg}}}}$ is added to the ECG signal $E_{s}\left(t\right)$ , yielding $E_{s}\left(t\right)+N\left(t\right)$ . The noise power $E_{N_{\text{avg}}}$ is derived from the signal power $E_{E_{\text{avg}}}$ and a specified Signal-to-Noise Ratio α , using $E_{N_{\text{avg}}}=10^{\left(E_{E_{\text{avg}}}-\alpha \right)/10}$ .

#### 5.1.2 Scaling

The ECG signal is scaled by a constant factor b>0 , yielding $b\cdot E_{s}\left(t\right)$ . This operation adjusts the signal amplitude uniformly.

#### 5.1.3 Temporal Inversion

This operation reverses the signal in time, transforming $E_{s}\left(t\right)$ into $E_{s}^{\prime }\left(t\right)$ , where the time indices are flipped from t=1,…,N to t=N,…,1 .

Although temporal inversion does not occur in real monitoring conditions, it remains a valuable self-supervised pretext task because it forces the model to reason about the directional structure of physiological waveforms. Reversing the signal disrupts its causal and morphological progression such as the systolic–diastolic sequence in PPG or the P–QRS–T order in ECG without altering its overall distribution, enabling the encoder to learn representations that are sensitive to true temporal dependencies rather than superficial signal features. In self-supervised learning, the goal is not to replicate realistic perturbations but to design surrogate tasks that elicit robust temporal feature learning. Temporal inversion has been widely adopted in time-series self supervised learning for this reason ((Zhang et al., 2023; Sarkar and Etemad, 2022)), providing a strong discriminative signal that helps the model internalize physiological timing relationships and ultimately supports more accurate ECG generation from PPG in the downstream GAN stage. Permutation: The signal is segmented into m parts $\left\{s_{i}\left(t\right)\right\}$ , which are randomly reordered to form a new sequence $E_{s_{p}}\left(t\right)$ , disrupting temporal order while preserving local signal structure.

#### 5.1.4 Negation

This transformation inverts the ECG signal $E_{s}\left(t\right)$ by multiplying it by −1 , yielding $-E_{s}\left(t\right)$ , which vertically flips the waveform and reverses signal polarity. It simulates polarity reversal, which may occur due to inverted sensor placement, such as with the Polar H10 device.

#### 5.1.5 Time-Warping

This technique alters the ECG signal $E_{s}\left(t\right)$ by stretching or compressing segments along the time axis using an interpolation-based function $F\left(E_{s}\left(t\right),k\right)$ , where k denotes the stretch factor and 1/k the compression factor. The signal is divided into m windows $\left\{s_{i}\left(t\right)\right\}$ , with randomly selected segments stretched and others compressed to preserve overall signal dynamics. The final signal T(t) is normalized in length via clipping or zero-padding, depending on whether m is even or odd.

#### 5.1.6 Transformation parameters

To ensure diverse signal transformations while preserving core ECG characteristics, we varied transformation parameters across broad ranges. For noise addition, signal-to-noise ratios (SNR) ranged from 2 to 45. Scaling factors ranged from 0.1 to 10. Permutation and time-warping used 2 to 40 segments, with time-warping stretch factors between 1.05 and 4. Temporal inversion and negation, which lack tunable parameters, were also included.

**FIGURE 4:**
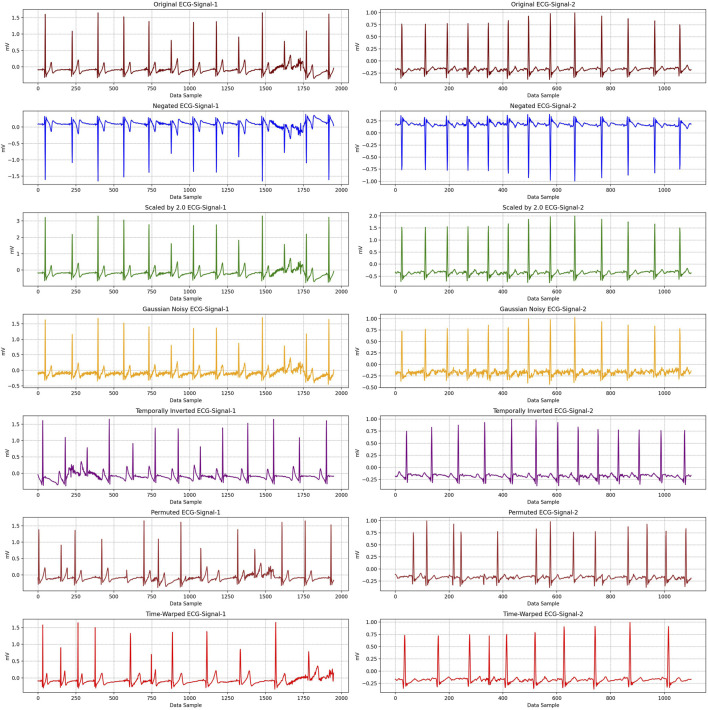
Transformed two samples of ECG signals from two participants for pretext tasks of self-supervised learning. Noise addition, scaling, temporal inversion, permutation, negation and time-warping were implemented to each signals, separately.

We should point out that these ranges were selected to generate a spectrum of signals—from near-original to substantially altered—capturing variations in heartbeat periodicity and waveform morphology (P-wave, QRS complex, T-wave). This enables the model to learn robust, generalizable spatio-temporal features without labeled data. Two sample signals from two different participants with these transformations are shown in Figure 4.

### 5.2 Datasets for pre-training

In addition to our original dataset and the MIMIC III dataset, we utilized three widely recognized PPG-ECG paired datasets to pre-train our proposed model: BIDMC (Pimentel et al., 2017), CAPNO (Karlen et al., 2013), and WESAD (Schmidt et al., 2018). These datasets were combined to support a multi-corpus pre-training strategy, enabling the model to learn from a diverse range of data encompassing variations in activity (e.g., working, walking, resting) and age (e.g., 29 children, 81 adults). The resulting dataset comprises 110 participants with a balanced gender distribution.

#### 5.2.2 BIDMC

Contains 8-min recordings from 53 adult ICU patients (mean age: 64.81 years; 32 females, 21 males), sampled at 125 Hz. Only ECG lead II was used.

#### 5.2.2 CAPNO

Includes 8-min recordings from 42 participants (29 children, median age 8.7; 13 adults, median age 52.4), with single-lead ECG and PPG sampled at 300 Hz.

#### 5.2.3 WESAD

Comprises data from 15 participants (mean age: 27.5), recorded during various activities. ECG was sampled at 700 Hz and PPG at 64 Hz, with session durations exceeding 1 hour.

### 5.3 Data pre-processing

Firstly, ECG and PPG data were synchronized/aligned. The alignment process here means the systolic peak of the PPG beat is exactly aligned with the R peak of the ECG beat. After this, given that the aforementioned datasets were collected at different sampling frequencies, the initial step involved re-sampling both ECG and PPG signals to a uniform sampling rate of 130 Hz using cubic spline interpolation technique. This approach was chosen to preserve at least the sampling rate of the ECG signal in our original dataset.

Raw ECG and PPG signals inherently contain various types and levels of noise, including power line interference, baseline wandering, and motion artifacts. In our study, motion artifacts were particularly prevalent, as participants frequently moved while engaging in VR gameplay. While it is essential to suppress noise components, it is equally critical to preserve the physiological signal content, particularly the energy distribution associated with ECG morphology and the slow heart rate related components in PPG. Therefore, appropriate band-pass filtering ranges were selected to retain diagnostically relevant information while removing unwanted noise. Specifically, ECG signals were filtered using a band-pass finite impulse response (FIR) filter with a passband of 0.5–45 Hz, ensuring preservation of P–QRS–T morphology and suppression of both baseline wander and high-frequency interference. Similarly, PPG signals were filtered with a band-pass Butterworth filter between 0.5–8 Hz to maintain morphological integrity related to cardiac pulsatility and slower hemodynamic variations. Additionally, a median non-linear filter was applied to both signals for removing motion artifacts and spikes, thereby producing smoother signals suitable for feeding to the GAN model. Subsequently, the filtered ECG and PPG signals were segmented into different segment windows (4, 8, 16, 32, 64, 96, 128, 160s), resulting in 130×n samples per window, with a 20% overlap between consecutive windows to ensure comprehensive peak detection. Finally, person-specific min-max normalization was performed on both the ECG and PPG segments, standardizing the data within the range of (−1, 1). After generation step, inverting min-max was applied to acquire signal with original scale.

### 5.4 Model training and fine-tuning

The model training task consist of two steps: 1- Multi-task self-supervised pre-training with aforementioned three datasets, 2- Full GAN model training and discriminator fine-tuning for Who is Alyx? and MIMIC III datasets, separately. To find the best hyperparameters for the model, we conducted a grid search covering 13,824 different model configurations per step for total training of models per dataset. The hyperparameters that led to the best classification results are shown in Table 1.

**TABLE 1:** The variables and their values that were used in the grid search to optimize the model’s hyperparameters and best performing values for the self-supervised pre-training and the discriminator fine-tuning for original (Who is Alyx?) and MIMIC III datasets.

Hyperparameters	Values	Self-supervised pre-training	Discriminator fine-tuning	
			Who is Alyx?	MIMIC III
Exponential decay $\beta_{1}$ , $\beta_{2}$	0.5, 0.9, 1, 3	3, 1	0.5, 0.9	0.9, 1
Gradient Penalty Scale Factor (λ)	4, 6, 30, 50	30	6	30
Fully Connected Layer	128, 256, 512	128, 128	512, 256	512, 256
Learning Rate	0.001, 0.002, 0.005	0.001	0.002	0.001
Dropout	0.2, 0.4, 0.5, 0.7	0.5, 0.4	0.4, 0.2	0.5, 0.2
Batch Size	64, 128	128	128	128

#### 5.4.1 Multi-task self-supervised pre-training

Our initial goal is to train the discriminator using the three aforementioned datasets from the literature, primarily aiming to learn robust features for generalization through a self-supervised approach. Following the pre-processing steps, for self-supervised signal transformation training, we randomly shuffled these three datasets, ensuring that the alignment between PPG and ECG pairs remained intact. The resulting segments were used for pretext tasks. Each segment was utilized to generate the six signal transformations described earlier.

**FIGURE 5:**
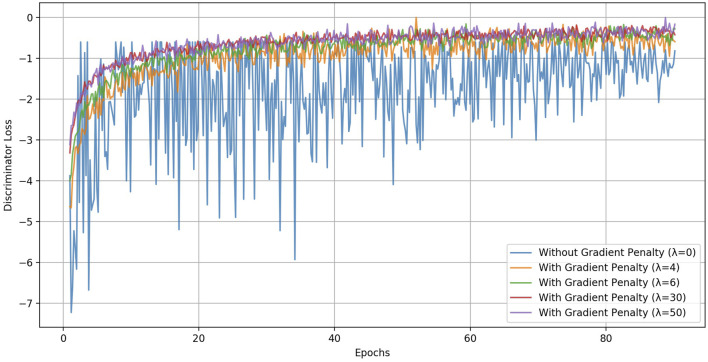
The impact of the gradient penalty coefficient (factor) λ as hyperparameter on the training of the discriminator on original dataset. It addresses the mode collapse issue and strengthen the model stability during training. It clearly demonstrate that the absence of the gradient penalty (λ=0) leads to notable instability in the training process, underscoring the importance of this parameter in ensuring robust and reliable model performance.

To facilitate this training as downstream task for the discriminator, we appended two fully connected layers to the end of the discriminator as task-specific layers. Both layers were set to a size of 128 and were followed by a Relu activation layer (Xu et al., 2015). We deliberately kept the fully connected layers simple and relatively shallow to effectively assess the capability of the self-supervised approach in learning robust and generalized ECG representations for the discriminator. This multi-task network was then trained on the six different signal transformation tasks with automatically generated pseudo-labels. The network was trained for 90 epochs with a batch size of 128 using the Adam optimizer (Kingma and Ba, 2014). 10-fold-cross validation was adopted to check the optimization performance on the training. To address the mode collapse issue and strengthen the model instability during training, we searched best performing gradient penalty scaling factor. Figure 5 shows how λ effects the model stability during training.

Subsequently, the weights of the discriminator’s transformer were frozen for use in the fine-tuning process.

#### 5.4.2 Training with original dataset and discriminator fine-tuning

Next, we trained our full GAN model, including both the discriminator and generator. Before initiating the training process of the model, the frozen weights from the discriminator’s Transformer were transferred for the GAN training with generator, as illustrated as ‘*transfer learning*’ in Figure 3. For the fine-tuning, fully connected layers with sizes of 512 and 256 (from 128 to 512 sized different combinations, see Table 1) with Relu activation layers (Xu et al., 2015) were added to the discriminator. This setup enabled the model to learn the general representations of the ECG patterns from the original dataset to effectively distinguish between real and generated (synthetic) segments. Each MIMIC III and Who is Alyx? dataset, along with the three previously mentioned datasets, was incorporated into the training phase of the GAN model.

To ensure reliable performance evaluation, we employed a leave-one-subject-out (LOSO) cross-validation strategy. Specifically, we performed 34-fold cross-validation by splitting the subjects from our original dataset into 34 groups, with each participant assigned to one group. For each iteration, the model was trained on 33 subjects and with other three datasets and tested on the remaining subject, allowing us to systematically evaluate the generalization capability of the model on participants that were never seen during training. Prior to each testing session, we randomly selected 10% of the training set as a validation set to ensure optimal model performance on the test set. This process also applied to MIMIC III dataset. Additionally, a time window analysis was conducted by segmenting the data into 4, 8, 16, 32, 64, 96, 128 and 160-s intervals (windows) to explore the temporal significance of deep features on the learning capacity of the models.

In total, data from 242 participants—comprising approximately 125 h of ECG-PPG segment pairs (55 h from Who is Alyx?, 8.25 h from MIMIC III, and 62 h from the pre-training datasets)—were utilized in the training process, yielding around 238k time-aligned segments. The model was trained using the Adam optimizer with the hyper-parameters listed in Table 1. Training was carried out over 90 epochs, with early stopping applied to prevent overfitting and ensure optimal performance.

Additionally, we also aimed to investigate the model’s capacity and the impact of excluding the self-supervised learning technique. In this scenario, the entire model, including the discriminator, was trained from-scratch using the same parameters as before, without freezing any layers during the training process. The training was conducted on a machine equipped with an Intel Core i9-13900K CPU, 128 GB of memory, and a NVIDIA RTX 4090 GPU. All models were implemented using the PyTorch 1.10 deep learning library.

## 6 Evaluation

Following the training phase, we evaluated the model’s performance using well-known metrics for ECG generation (Sarkar and Etemad, 2020; Vo et al., 2021), ensuring a fair comparison with related works:

### 6.1 Fréchet Distance (FD)

FD quantifies the spatial and sequential similarity between the real and generated ECGs. A lower FD value, closer to zero, indicates a higher similarity between the real ECG and its synthesis. FD is defined as:

$$FD=\underset{Q}{\min}\left(\underset{i\in Q}{\max}\left(d\left(y_{\text{ECG}i},y_{r\text{ECG}i}\right)\right)\right)$$

where Q=[1,m] and d(∗) represents the Euclidean distance between corresponding points on the ECG and synthesis curves.

### 6.2 Root mean squared error (RMSE)

The Root Mean Squared Error (RMSE) measures the discrepancy between the observed values of an ECG signal and its reconstructed values by aggregating the squared differences between them. It is conventionally quantified with millivolts (mV) for ECG signals. A smaller RMSE value, closer to zero, indicates a more accurate reconstruction. RMSE is defined as:

$$RMSE=\sqrt{\frac{1}{N}\overset{N}{\underset{i=1}{\sum }}{\left(y_{\text{ECG}i}-y_{r\text{ECG}i}\right)}^{2}}$$

### 6.3 Pearson’s correlation coefficient (ρ)

Pearson’s correlation coefficient (ρ) is used to assess the degree of linear relationship between an original ECG signal and its reconstructed counterpart. The coefficient ρ ranges from −1 to 1, where the absolute value indicates the strength of the correlation, and the sign indicates the direction (positive or negative) of the relationship. Closer to 1 indicates a strong positive correlation between signals. The correlation coefficient is computed as follows:

$$\rho =\frac{{\left(y_{\text{ECG}}-{\bar{y}}_{\text{ECG}}\right)}^{T}\left(y_{r\text{ECG}}-{\bar{y}}_{r\text{ECG}}\right)}{\|y_{\text{ECG}}-{\bar{y}}_{\text{ECG}}{\|}_{2}\|y_{r\text{ECG}}-{\bar{y}}_{r\text{ECG}}{\|}_{2}}$$

$y_{\text{ECG}}$ and $y_{r\text{ECG}}$ denote the original and generated ECG, respectively, and $\|\cdot {\|}_{2}$ is the Euclidean norm.

### 6.4 Mean Absolute Error for Heart Rate ( ${\text{MAE}}_{\text{HR}}$ )

Heart rate (HR) is computed from the R-R interval as

$$\text{HR }\left(\text{bpm}\right)=\frac{60}{\text{R}-\text{R Interval }\left(\text{seconds}\right)}$$

${\text{MAE}}_{\text{HR}}$ (in BPM) is calculated between the estimated HR from a given ECG or PPG signal $\left({\text{HR}}_{Q}\right)$ and the ground-truth HR $\left({\text{HR}}_{GT}\right)$ as follows:

$${\text{MAE}}_{\text{HR}}\left(Q\right)=\frac{1}{N}\overset{N}{\underset{i=1}{\sum }}|{\text{HR}}_{GT,i}-{\text{HR}}_{Q,i}|,$$

where N represents the number of segments for which HR measurements were obtained. To evaluate the performance of our model, we measure ${\text{MAE}}_{\text{HR}}\left(E^{\prime }\right)$ , where $E^{\prime }$ is the ECG generated by the model. These MAE values are compared to ${\text{MAE}}_{\text{HR}}\left(P\right)$ , where P represents the input PPG signals. This comparison allows us to assess the model’s performance in generating ECG signals that closely match the ground-truth, in this case real ECG, HR values. As expected, ${\text{MAE}}_{\text{HR}}\left(E^{\prime }\right)$ value should be lower than ${\text{MAE}}_{\text{HR}}\left(P\right)$ value, ideally approaching to zero. We utilized two widely recognized algorithms for peak detection from ECG (Hamilton, 2002) and PPG (Elgendi et al., 2013) signals for heart rate calculation. Using those two algorithms is a consistent criteria to evaluate the MAE metric since they have been used in similar works (Tang et al., 2022; Sarkar and Etemad, 2020).

## 7 Results

In this section, we present quantitative and qualitative results of our proposed Transformer-based GAN model for ECG generation. The impact of the self-supervised approach and different segment lengths is evaluated using mean values of RMSE (mV), ρ , FD, and ${\text{MAE}}_{\text{HR}}$ metrics under LOSO approach for both MIMIC III and original (Who is Alyx?) datasets, as shown in Table 2, 3, respectively.

**TABLE 2:** Our proposed PPG-to-ECG generation model was evaluated on the MIMIC III dataset as benchmark dataset. Performance was assessed across varying window lengths, with and without self-supervised learning, using LOSO mean values (and their standard deviations) of RMSE (mV), FD, ρ , ${\text{MAE}}_{\text{HR}}\left(E^{\prime }\right)$ , and ${\text{MAE}}_{\text{HR}}\left(P\right)$ .

Segment length (s)	Method	RMSE (mV)	FD	ρ	${\text{MAE}}_{\text{HR}}\left(E^{\prime }\right)$	${\text{MAE}}_{\text{HR}}\left(P\right)$
4	w/o Self-Supervised	0.389±0.017	0.691±0.020	0.756±0.013	2.37±0.11	7.23±0.21
	Self-Supervised	0.364±0.015	0.642±0.018	0.858±0.010	2.16±0.09	
8	w/o Self-Supervised	0.352±0.014	0.651±0.018	0.801±0.012	2.12±0.10	6.78±0.20
	Self-Supervised	0.326±0.013	0.612±0.017	0.874±0.009	1.94±0.08	
16	w/o Self-Supervised	0.232±0.011	0.442±0.015	0.808±0.011	1.93±0.09	6.23±0.18
	Self-Supervised	0.197±0.010	0.421±0.014	0.894±0.008	1.69±0.07	
32	w/o Self-Supervised	0.198±0.009	0.431±0.014	0.875±0.010	1.58±0.08	6.01±0.18
	Self-Supervised	0.185±0.008	0.409±0.012	0.927±0.008	1.14±0.06	
64	w/o Self-Supervised	0.181±0.008	0.405±0.013	0.911±0.009	1.28±0.07	5.87±0.17
	Self-Supervised	0.175±0.008	0.386±0.012	0.940±0.007	1.08±0.06	
96	w/o Self-Supervised	0.172±0.007	0.387±0.012	0.941±0.008	1.09±0.06	5.45±0.16
	Self-Supervised	0.168±0.007	0.368±0.011	0.952±0.007	0.88±0.05	
128	w/o Self-Supervised	0.186±0.008	0.455±0.014	0.895±0.009	1.45±0.07	5.67±0.18
	Self-Supervised	0.187±0.008	0.422±0.013	0.928±0.008	1.15±0.06	
160	w/o Self-Supervised	0.298±0.012	0.517±0.016	0.863±0.010	1.67±0.08	5.82±0.19
	Self-Supervised	0.242±0.011	0.442±0.014	0.906±0.009	1.38±0.07	

**TABLE 3:** We present PPG-to-ECG generation results using our Transformer-based GAN model on Who is Alyx? dataset. LOSO mean values (and their standard deviations) are reported with and without self-supervised learning, evaluated using RMSE, FD, ρ , ${\text{MAE}}_{\text{HR}}\left(E^{\prime }\right)$ , and ${\text{MAE}}_{\text{HR}}\left(P\right)$ across various window lengths.

Segment length (s)	Method	RMSE (mV)	FD	ρ	${\text{MAE}}_{\text{HR}}\left(E^{\prime }\right)$	${\text{MAE}}_{\text{HR}}\left(P\right)$
4	w/o Self-Supervised	0.541±0.027	0.904±0.031	0.661±0.020	7.12±0.42	12.56±0.45
	Self-Supervised	0.478±0.024	0.782±0.029	0.786±0.018	7.03±0.39	
8	w/o Self-Supervised	0.507±0.025	0.729±0.028	0.772±0.019	5.68±0.36	11.34±0.42
	Self-Supervised	0.437±0.022	0.667±0.027	0.842±0.017	4.83±0.33	
16	w/o Self-Supervised	0.412±0.023	0.673±0.026	0.788±0.018	5.12±0.34	10.43±0.40
	Self-Supervised	0.344±0.021	0.606±0.025	0.852±0.016	3.41±0.30	
32	w/o Self-Supervised	0.319±0.022	0.589±0.025	0.798±0.018	4.89±0.33	10.21±0.39
	Self-Supervised	0.280±0.020	0.534±0.024	0.874±0.015	2.95±0.28	
64	w/o Self-Supervised	0.273±0.020	0.552±0.024	0.843±0.017	3.74±0.31	10.27±0.38
	Self-Supervised	0.220±0.018	0.493±0.022	0.907±0.015	2.84±0.26	
96	w/o Self-Supervised	0.316±0.023	0.623±0.027	0.829±0.018	4.01±0.33	10.33±0.39
	Self-Supervised	0.252±0.021	0.586±0.025	0.854±0.017	3.03±0.27	
128	w/o Self-Supervised	0.323±0.023	0.648±0.028	0.793±0.019	4.24±0.34	10.39±0.40
	Self-Supervised	0.267±0.022	0.591±0.026	0.839±0.017	3.13±0.28	
160	w/o Self-Supervised	0.351±0.025	0.682±0.029	0.778±0.020	4.55±0.36	10.42±0.41
	Self-Supervised	0.300±0.023	0.654±0.028	0.831±0.018	3.34±0.29	

We also evaluated the model’s performance in the absence of self-supervised learning. As shown in Table 2, 3, the self-supervised approach yielded significantly better results across all evaluated metrics, underscoring its effectiveness in the training process. Additionally, a time window analysis was performed by segmenting the data into 4, 8, 16, 32, 64, 96, 128 and 160-s intervals to investigate the temporal influence of deep features on the models’ learning capacity. To assess the impact of different window sizes on our approach, we measured ${\text{MAE}}_{\text{HR}}\left(E^{\prime }\right)$ values across the same window lengths used as input for the model. As shown in Table 3, all metrics significantly improved as the window length increased up to 64 s for Who is Alyx?. A strong correlation between the generated ECG signal and the ground-truth was observed, with an RMSE of 0.22 mV and a ρ value of 0.907 for the 64-s window. As shown in Table 2, we acquired the best result with 96-s windows for the MIMIC III with an RMSE of 0.168 mV and a ρ value of 0.952. However, a slight degradation in metric values was observed starting from the 96-s from Who is Alyx? and 128 s from MIMIC III, with further declines until 160-s windows. This indicates the model’s difficulty in effectively capturing time-series patterns for accurate reconstruction over longer intervals.

Overall, MIMIC III consistently yielded lower ${\text{MAE}}_{\text{HR}}$ and higher correlation values compared to Who is Alyx?, which is attributed to the controlled clinical environment of MIMIC III, in contrast to the motion-rich, real-world settings captured in the Who is Alyx? dataset—an expected and informative distinction.

**FIGURE 6:**
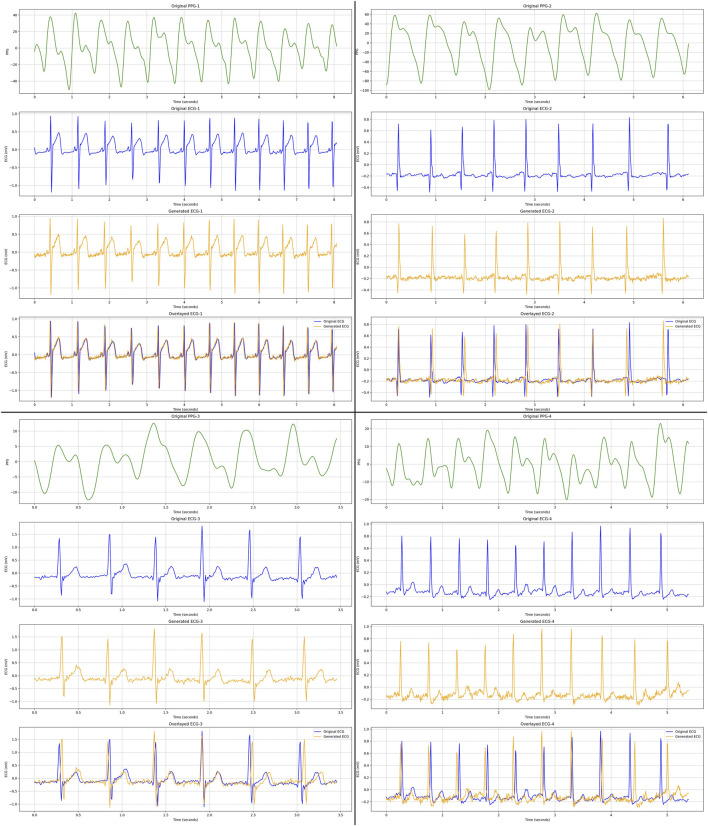
We present four distinct ECG samples (with different colors) generated by our proposed best performed Transformer-based GAN model on Who is Alyx? dataset. These four samples illustrate cases with minimal motion distortion, providing a clear representation and demonstrating the robust generation capability of the model for ECG signals. For each PPG sample (green color), corresponding real ECG (blue color), generated ECG (yellow color) and overlayed (original and generated) ECG signal (blue + yellow color) samples are displayed, respectively.

**FIGURE 7:**
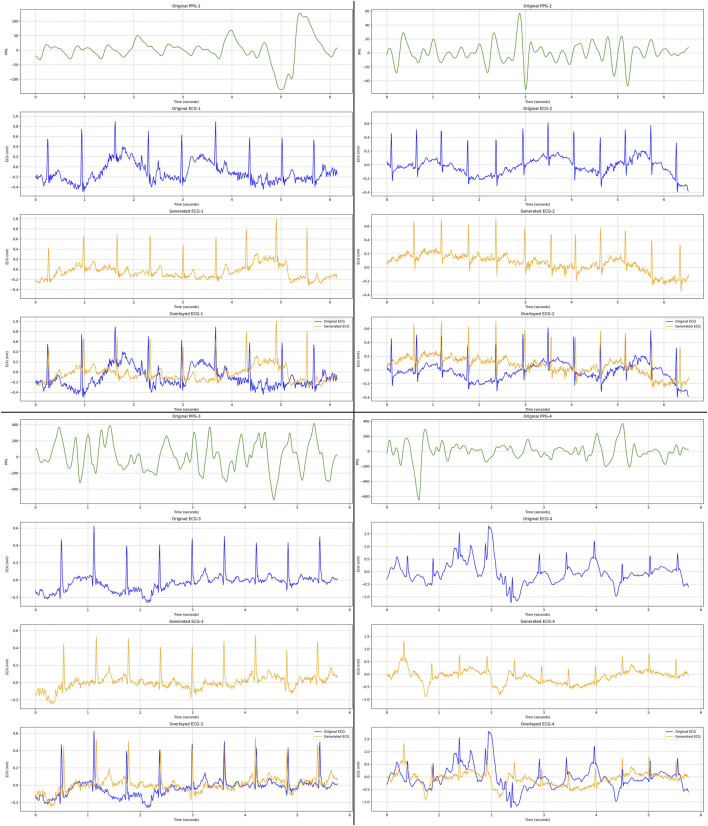
We present four distinct ECG samples (with different colors) generated by our proposed best performed Transformer-based GAN model on Who is Alyx? dataset. These four samples specifically highlight cases with motion artifacts, demonstrating the model’s performance under challenging conditions involving signal distortion. For each PPG sample (green color), corresponding real ECG (blue color), generated ECG (yellow color) and overlayed (original and generated) ECG signal (blue + yellow color) samples are displayed, respectively.

**FIGURE 8:**
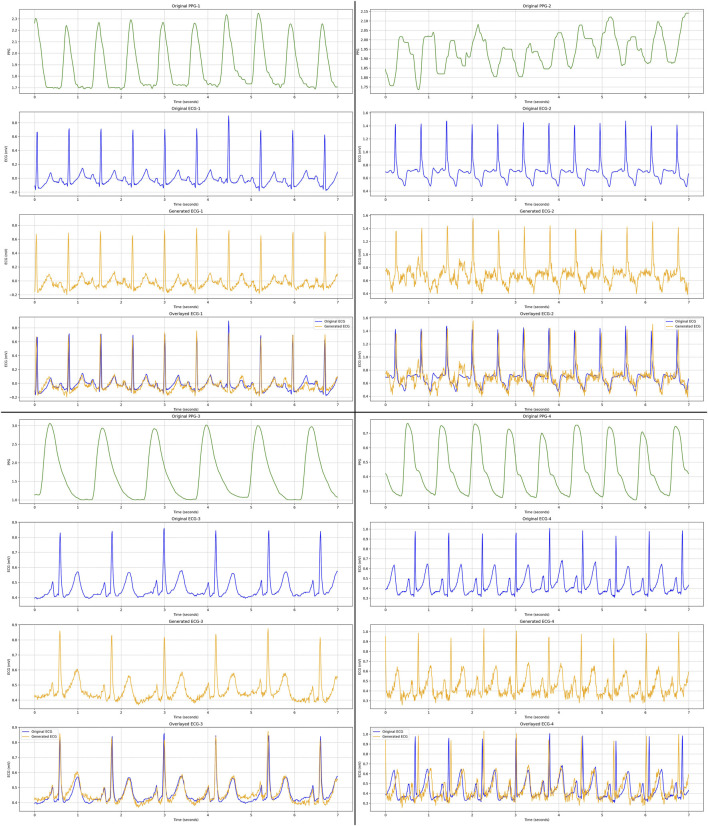
We present four distinct ECG samples (with different colors) generated by our proposed best performed Transformer-based GAN model on MIMIC III dataset. Comparing to Who is Alyx? dataset, MIMIC III has less motion artefacts and emotional changes. For each PPG sample (green color), corresponding real ECG (blue color), generated ECG (yellow color) and overlayed (original and generated) ECG signal (blue + yellow color) samples are displayed, respectively.

As qualitative results, Figures 6, 7 display several samples of ECG signals generated by our best performed model for Who is Alyx?, illustrating the model’s ability to reconstruct the shape of the original ECG signals from corresponding 64-second-segments of PPG inputs. Figure 8 shows the samples generated ECG signals on MIMIC III with 96-second-segments of PPG inputs.

### 7.1 Distributional comparison between generated and real ECG

While our proposed model demonstrates strong performance in generating ECG from PPG, it is also important to examine potential distributional differences between the generated and real ECG data. To this end, we utilized our Transformer-based discriminator to extract high-dimensional feature embeddings, following techniques commonly used for ECG feature representation (Singh and Krishnan, 2023). Specifically, we removed the final fully connected layer and obtained a 512-dimensional embedding from the preceding fully connected layer, effectively repurposing the discriminator as an autoencoder-like feature extractor capable of capturing meaningful sequential representations. For this analysis, we randomly generated 200 ECG samples from the test set for each dataset using their respective best-performing hyperparameters and segment lengths. Each real and generated ECG sample produced a 512-dimensional embedding during a forward pass through the discriminator.

**FIGURE 9:**
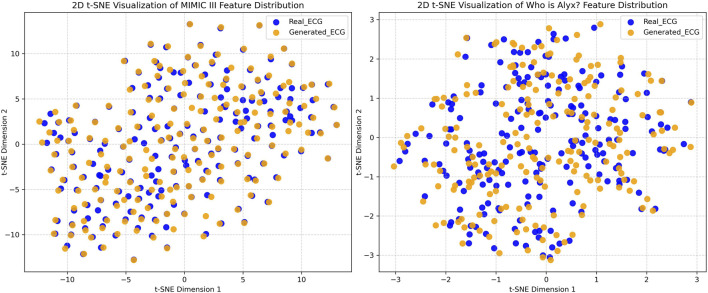
Two-dimensional t-SNE projections of learned feature embeddings for 200 real and 200 generated ECG samples for MIMIC III (left) and Who is Alyx (right) datasets.

To visualize and assess distributional similarities or divergences between the real and generated data, we applied t-distributed Stochastic Neighbor Embedding (t-SNE), a nonlinear dimensionality reduction technique that projects high-dimensional data into a low-dimensional (2D or 3D) space while preserving local neighborhood structure. We present the resulting 2D t-SNE scatter plots in Figure 9. The perplexity and learning rate parameters of t-SNE were set to 50 and 600, respectively, for both datasets.

Furthermore, to quantitatively evaluate the similarity between the distributions of real and generated ECGs, we computed two complementary two-sample statistical divergence measures: Maximum Mean Discrepancy $\left(MMD^{2}\right)$ (Gretton et al., 2012) and Energy Distance $\left(ED^{2}\right)$ (Székely and Rizzo, 2013), using the learned feature embeddings. Both metrics provide non-parametric assessments of distributional differences by measuring discrepancies in pairwise distances between samples. In addition, we performed hypothesis testing under the null hypothesis $\left(H_{0}\right)$ that the real and generated samples originate from the same distribution, using a significance level of 0.05. The resulting $MMD^{2}$ , $ED^{2}$ , and permutation-based p-values for each dataset are reported in Table 4.

**TABLE 4:** Two-sample squared Maximum Mean Discrepancy $\left(MMD^{2}\right)$ and squared Energy Distance $\left(ED^{2}\right)$ values, along with permutation test p-values for the null hypothesis $\left(H_{0}\right)$ .

Dataset	$MMD^{2}$	p-value	$ED^{2}$	p-value
MIMIC III	0.011±0.002	0.84	0.032±0.005	0.73
Who is Alyx?	0.015±0.004	0.78	0.043±0.011	0.69

Ideally, both $MMD^{2}$ and $ED^{2}$ values should approach zero, indicating minimal divergence and substantial overlap between the two distributions in feature space. The obtained scores indeed suggest that the average pairwise distances between the real and generated feature sets are highly similar. The permutation p-values (>0.05) further indicate insufficient statistical evidence to reject $H_{0}$ , implying that the distributions are indistinguishable under these tests.

Notably, the divergence scores for the Who is Alyx? dataset are slightly higher than those for MIMIC III, reflecting the greater presence of motion artifacts and noise, which can partially obscure the extraction of stable ECG-specific features. This observation aligns with the t-SNE visualizations in Figure 9: while the MIMIC III feature embeddings for real and generated ECGs exhibit strong overlap, the Who is Alyx? feature embeddings display minor cluster separation, indicating subtle distributional differences. These results are consistent with the observed morphological similarity between real and generated ECG signals, while also highlighting the inherent challenges posed by motion-distorted data.

## 8 Practical implementation: AF detection with deep learning

To demonstrate the practical utility of the proposed model and assess whether the generated ECG signals behave similarly to real ECG in downstream clinical tasks, we performed an Atrial Fibrillation (AF) detection experiment using the MIMIC PERform AF dataset. AF is typically characterized in ECG recordings by the absence of P-waves, irregular baseline fluctuations between QRS complexes, and increased beat-to-beat variability. Given the adequacy of both dataset size and segment duration, we implemented two widely adopted deep learning architectures, Bidirectional LSTM (Bid-LSTM) and a hybrid CNN + LSTM model which have shown strong performance across numerous sequence classification tasks in the literature. These models were trained to classify each ECG segment as either AF or non-AF (normal sinus rhythm). To ensure fair optimization and robust performance, we conducted a grid search to identify the best-performing hyperparameters for each architecture, using the same parameter search space defined in Table 1.

### 8.1 Bid-LSTM model

Bid-LSTM model consists of five layers: two LSTM layers with 128 hidden units each, a dropout layer with a dropout probability of 0.5, and two fully connected layers with 256 units. The input to the first LSTM layer is a tensor of shape (batch size, time window, features). Each LSTM layer outputs a tensor of shape (batch size, LSTM hidden size), corresponding to the final hidden states after processing the full temporal sequence. Following the dropout operation, the resulting representation is passed through the two fully connected layers, each utilizing a ReLU activation function (Nair and Hinton, 2010). Unlike a standard unidirectional LSTM, the Bid-LSTM architecture concatenates the hidden states from both forward and backward temporal passes. This bidirectional processing enables the network to capture dependencies from both past and future time steps, thereby improving its ability to learn discriminative temporal features relevant for AF classification.

### 8.2 CNN + LSTM model

The hybrid CNN–LSTM architecture comprises ten layers: two 1D convolutional layers (Conv1D), two max-pooling layers, a dropout layer, followed by the same five layers used in the Bid-LSTM model (two LSTMs, one dropout layer, and two fully-connected layers). The input to the first Conv1D layer has the shape (batch size, time window, features). Both Conv1D layers use a kernel size of 4, ReLU activation, and a number of filters equal to the input dimensionality expected by the subsequent LSTM layers. Each Conv1D layer is followed by max pooling with a pool size of 2 and a stride of 2, reducing the temporal dimension while preserving salient local temporal features. After the second pooling layer, the output tensor has the form (batch size, reduced time window, LSTM input size), making it directly compatible with the LSTM layers. The subsequent LSTM and fully connected layers mirror the configuration used in the Bid-LSTM model, enabling the hybrid network to leverage both local convolutional feature extraction and long-range temporal modeling.

### 8.3 Data generation and training

As a preliminary step, we fine-tuned the discriminator of our proposed model, as described in Section 5.4.2, using ECG data from 7 participants (4 AF, 3 non-AF) to capture AF-specific morphological patterns. For the AF detection task, the dataset was partitioned into training and testing sets at approximately a 0.68/0.32 ratio, resulting in 19 participants (10 AF, 9 non-AF) for training and 9 participants (5 AF, 4 non-AF) for testing. After applying min–max normalization, 30-s segments were used as inputs for each classifier. Using the PPG data of all 28 train + test participants, we generated corresponding ECG data with our proposed PPG-to-ECG model to create both training and testing sets for evaluation. The testing participants were deliberately selected to differ from those in the training set to assess subject-independent generalization performance. Additionally, 10% of the training data was randomly allocated as a validation set for hyperparameter optimization. The Bid-LSTM model was trained for 70 epochs and the CNN + LSTM model for 110 epochs, both with a batch size of 128 and the Adam optimizer (Kingma and Ba, 2014).

### 8.4 Evaluation and results

#### 8.4.1 Data mixing for training

To examine the contribution of generated ECG data in the AF detection task, we systematically varied the proportion of generated data used during training. We first trained the classifiers using only real ECG data (100%) as a baseline. Subsequently, generated ECG samples were incorporated in increments of 25% while maintaining a constant total training set size. Performance was assessed using accuracy, precision, recall (sensitivity), and F1-score on the real test set. The results summarizing the models’ learning behavior across different mixing ratios are presented in Table 5.

**TABLE 5:** AF detection performance (accuracy, precision, recall/sensitivity, and F1-score) of the CNN + LSTM and Bid-LSTM models across varying proportions of real and generated ECG data used in the training set.

Train set partition (%)		Model	Accuracy	Precision	Recall	F1-score
Real	Gen					
100	0	CNN + LSTM	0.922	0.916	0.946	0.931
		Bid-LSTM	0.951	0.949	0.967	0.957
75	25	CNN + LSTM	0.908	0.913	0.915	0.903
		Bid-LSTM	0.938	0.941	0.923	0.928
50	50	CNN + LSTM	0.884	0.879	0.892	0.889
		Bid-LSTM	0.913	0.921	0.902	0.921
25	75	CNN + LSTM	0.862	0.854	0.872	0.867
		Bid-LSTM	0.908	0.913	0.898	0.913
0	100	CNN + LSTM	0.858	0.863	0.868	0.861
		Bid-LSTM	0.892	0.901	0.897	0.899

#### 8.4.2 Testing with generated test data

**FIGURE 10:**
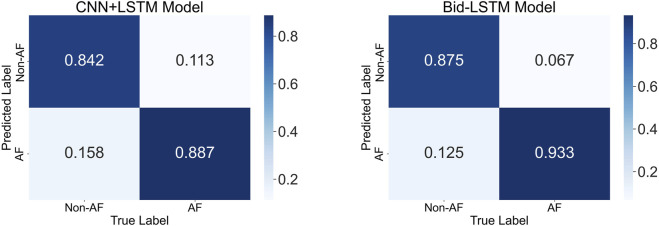
Confusion matrices illustrating AF detection performance on the generated ECG test set for the CNN + LSTM and Bid-LSTM models.

To evaluate whether the generated ECG signals can function as reliable surrogates for real ECG in practical deployment scenarios, we trained the AF classifiers exclusively on real ECG data and tested them on the generated ECG data generated for the test participants. This mirrors the real-world use case where a PPG-to-ECG model would supply the ECG input for downstream diagnostic algorithms. The resulting performance metrics are reported in Table 6. Confusion matrices for the best-performing training configuration are shown in Figure 10.

**TABLE 6:** AF detection performance (accuracy, precision, recall, and F1-score) of the CNN + LSTM and Bid-LSTM models evaluated exclusively on the generated ECG test set.

Model	Accuracy	Precision	Recall	F1-score
CNN + LSTM	0.864	0.849	0.887	0.868
Bid-LSTM	0.906	0.882	0.933	0.907

#### 8.4.3 Performance summary

Across all training and testing conditions, the Bid-LSTM model consistently outperformed the CNN + LSTM model. Its bidirectional structure, which processes information from both past and future temporal states, provided a clear advantage in capturing the sequential dependencies characteristic of AF rhythms, an ability that the CNN + LSTM architecture struggled to match.

Using only real ECG for both training and testing, the Bid-LSTM achieved the best performance with 95.1% accuracy and 95.7% F1-score, closely aligning with the results reported in (John et al., 2025) (95.9% accuracy). As the proportion of generated ECG data increased, accuracy declined by approximately 5.9%, which was expected due to the accumulating deviation from real data morphology. Nonetheless, training with only generated ECG data still yielded an accuracy of 89.2%, demonstrating strong diagnostic utility. Importantly, when evaluating solely on generated test ECG signals, the classifier exhibited good performance with 90.6% accuracy using Bid-LSTM, comparable to testing on real ECG, indicating that the generated data produced by our model retain the salient AF-related patterns necessary for reliable automated diagnosis. These results provide compelling empirical evidence that PPG-derived ECG from our model can serve as a viable input for downstream cardiovascular disease detection applications.

### 8.5 Measurement of baseline fluctuations

Baseline fluctuations in ECG signals play a crucial role in clinical diagnosis, particularly for conditions such as arrhythmias, ischemia, and conduction abnormalities. Therefore, it is essential to evaluate how closely the generated ECG baseline follows the real ECG baseline. To investigate this, we conducted two complementary analyses:

#### 8.5.1 Baseline evaluation via QRS removal

**FIGURE 11:**
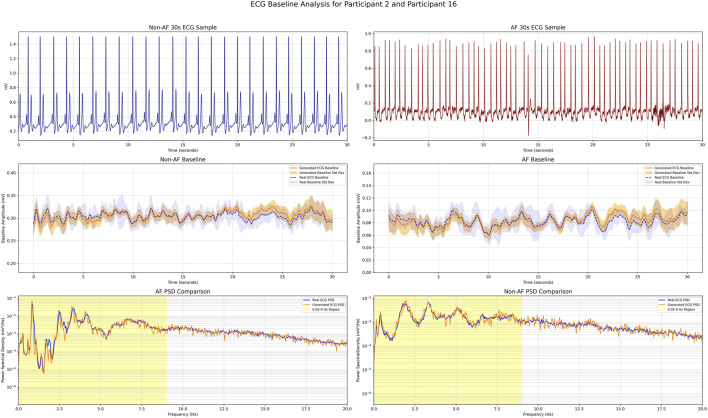
Qualitative comparison of real and generated ECG signals for two participants (AF and non-AF) from the MIMIC PERform AF Dataset. Top row: Representative 30-s segments of AF and non-AF ECG signals. Middle row: Baseline fluctuation profiles of real and generated ECGs from the full 20-min recordings, shown with mean values and standard deviations. Bottom row: Power spectral density (PSD) plots for AF and non-AF ECG signals, highlighting the 0.05–9 Hz frequency band relevant for baseline characterization and AF detection.

We first applied the Pan–Tompkins QRS detection algorithm (Pan and Tompkins, 1985) to identify and remove the QRS complexes from both real and generated ECG signals, isolating the baseline components (including P- and T-wave regions). The remaining non-QRS samples were linearly interpolated to obtain a continuous approximation of the underlying baseline morphology. This approach also allowed us to assess the generative model independently of heart-rate-related metrics, which are primarily determined by the QRS complex. Subsequently, we computed RMSE (mV), FD, and ρ between the real and generated baseline data for all test samples, with results summarized in Table 7. To provide qualitative insight, baseline fluctuation patterns from one AF and one non-AF participant are visualized in Figure 11.

**TABLE 7:** Baseline analysis of real and generated ECG signals from the test set of the MIMIC PERform AF Dataset. The first three columns present baseline comparisons using mean RMSE, FD, and ρ metrics. The final column reports the difference in band-limited (0.05–9 Hz) total spectral power $\left(mV^{2}\right)$ between real and generated ECGs.

MIMIC PERform AF dataset	RMSE (mV)	FD	ρ	Total band power $\left(mV^{2}\right)$
Non-AF ECG	0.174±0.024	0.365±0.073	0.949±0.121	0.014±0.004
AF ECG	0.187±0.054	0.387±0.091	0.921±0.153	0.018±0.006

#### 8.5.2 Spectral analysis of baseline dynamics

To further evaluate whether the generated ECG preserves the frequency characteristics of baseline fluctuations, we performed power spectral density (PSD) analysis using the Welch periodogram method (Welch, 1967). The analysis focused on the 0.05–9 Hz range, which encompasses two diagnostically relevant bands: (i) 0.05–1 Hz, associated with baseline wandering and slow morphological variations, and (ii) 3–9 Hz, where atrial fibrillation–related fibrillatory activity and the absence of organized P-waves are typically observed (Bollmann et al., 1998). Band-limited total spectral power was computed for real and generated ECGs in both AF and non-AF classes, and the results are reported in Table 7. PSD comparisons for two representative participants (one AF, one non-AF) are shown in Figure 11.

Overall, both quantitative and qualitative results demonstrate that the generated ECGs closely follow the baseline dynamics of real ECGs across AF and non-AF conditions. These dynamics are maintained consistently in both the time and frequency domains. While AF reconstruction shows slightly lower similarity scores, likely due to the highly variable and patient-specific morphology of AF baseline characteristics, the deviations remain within an acceptable range. Importantly, the fidelity of the generated baseline was sufficient to yield strong AF detection performance, supporting the practical viability of the generated data.

## 9 Discussion

We have demonstrated that the utilization of PPG signals from unobtrusive wearable devices as a simple setup, combined with appropriate GAN model and a supportive self-supervised learning, yields excellent results in synthesis ECG signals. Our proposed approach involves the use of a Transformer-based GAN model in conjunction with self-supervised signal transformation technique, achieving 0.22 mV RMSE value and 0.907 Pearson correlation coefficient (ρ) value (see Table 3) on Who is Alyx?. Also, our study shows that the ECG generated by our Transformer-based GAN model provides more reliable heart rate measurements compared to the original input PPG, reducing the error from 10.27 BPM (measured from the PPG) to 2.84 BPM (measured from the generated ECG) with 72.4% for 64-s window segments. Furthermore, we utilized MIMIC III dataset as baseline an achieved of 0.168 mV RMSE and 0.952 ρ value and %83.9 error reduction on this dataset. This outperforms previous works employing PPG data as input for ECG synthesis, including complex signal processing approaches and machine/deep learning techniques on this dataset.

### 9.1 Comparison with similar works from literature

A comparison with recent state-of-the-art studies that are most similar to our PPG-to-ECG generation work, particularly those employing GAN or deep learning models, is presented in Table 8. (Zhu et al., 2021). used DCT approach to acquire the relationship between PPG-ECG pairs. However, their approach demonstrated limited generalization to previously unseen participants and required extensive signal transformation and segmentation steps, rendering it unsuitable for end-to-end deployment. In terms of dataset diversity, the data used in (Zhu et al., 2021; Tian et al., 2023) were acquired in controlled clinical settings and lack motion artifacts or variability from daily-life activities. As such, the robustness and applicability of their models to real-world scenarios involving movement and noise remain uncertain. (Sarkar and Etemad, 2020). proposed CycleGAN-based solution for ECG generation. However, their model exhibited poor performance on the cases of involving noisy and motion artifacts. Furthermore, they reported lower ${\text{MAE}}_{\text{HR}}\left(P\right)$ values (9.74) in their study compared to ours. This is also a strong indication that our dataset contains substantial motion artifacts, which distorts PPG signals, thereby increasing the difficulty of accurate heart rate calculation. Also, they reported 70% error reduction and we outperformed their result with 83.9% on similar clinical dataset. Additionally, works such as (Vo et al., 2021; Zhu et al., 2021; Tang et al., 2022) partitioned their datasets into training and testing sets without separating participants. This practice raises concerns in machine learning research, as evaluating a model’s performance on unseen participants is crucial for assessing generalization. Consequently, their reported results may not reliably reflect the model’s capability in real-world deployment or across new individuals.

**TABLE 8:** Comparative summary of state-of-the-art ECG signal generation studies that utilize PPG as direct input, specifically to their GAN or deep learning models. Our results are presented alongside these studies for reference. The abbreviation “NR” indicates that the corresponding metric was not reported.

Work	Setup	Dataset	Segment length (s)	Methods	RMSE (mV)	ρ
Sarkar and Etemad (2020)	Various PPG-ECG Setup	DALIA, BIDMC, CAPNO, WESAD	4	CycleGAN-based	0.396	NR
Zhu et al. (2021)	Medical PPG-ECG, Empatica	MIMIC III, Self-collected	24 beats	DCT	0.599, 0.447	0.790, 0.895
Tang et al. (2022)	Medical PPG-ECG Setup	MIMIC III	48	Bil-LSTM	0.403	0.904
Vo et al. (2021)	Medical PPG-ECG Setup	MIMIC II	3	Wasserstein GAN	0.238	0.835
Tian et al. (2023)	Medical PPG-ECG Setup	MIMIC III	3	Dictionary Learning	0.39	0.88
Lan (2023)	Medical PPG-ECG Setup	BIDMC	4	Patch-based Transformer	0.29	NR
Abdelgaber et al. (2023)	Medical PPG-ECG Setup	MIMIC II	1 beat	LSTM-based Autoencoder	0.35	0.923
Guo et al. (2024)	Medical PPG-ECG Setup	MIMIC III	3	UNet-BidLSTM	0.077	0.861
Shome et al. (2024)	Various PPG-ECG Setup	BIDMC, MIMIC III	4	Diffusion Model (UNet)	0.24, 0.22	NR
Vo et al. (2024)	Medical PPG-ECG Setup	MIMIC III	4	State-Space Attention	0.076	0.847
Belhasin et al. (2025)	Medical PPG-ECG Setup	MIMIC III	8	UA-P2E	0.222	NR
Our work	Polar H10, Empatica	Who is Alyx?, MIMIC III	64	Self-Supervised Transf-GAN	0.22, 0.168	0.907, 0.952

A review of recent studies (2023–2024) on PPG-to-ECG signal generation indicates meaningful progress (Table 8); however, several critical limitations remain, particularly concerning subject-independent generalization, robustness to motion-induced artifacts, and reliance on highly curated or heavily preprocessed datasets. For example, (Shome et al., 2024), proposed the Region-Disentangled Diffusion Model, a diffusion-based U-Net architecture that reconstructs ECG segments using region-specific noise injection. Although this method achieved RMSE values of 0.22 on MIMIC and 0.24 on BIDMC, the framework depends on carefully controlled noise injection across predefined waveform segments. Moreover, the substantial performance drop in AF detection (accuracy of only 0.65) using the generated ECG suggests diminished reliability under conditions involving uncontrolled motion artifacts. Similarly, (Vo et al., 2024), introduced an attention-based deep state-space model for PPG-to-ECG translation. Despite its conceptual strengths, the approach requires explicit peak detection and noise-injection procedures, raising concerns about its viability as a fully end-to-end system in realistic environments. Furthermore, the relatively low ρ values reported indicate suboptimal waveform reconstruction fidelity, a key metric for evaluating generation success.

Transformer-based approaches have also recently emerged. For instance, (Lan, 2023), employed a shifted patch-based attention mechanism enhanced with multimodal digital biomarkers. While innovative, this design requires manually engineered patch structures and multiple signal modalities, substantially increasing computational complexity and limiting applicability in wearable or real-time deployment scenarios. Likewise, (Belhasin et al., 2025), proposed a diffusion model incorporating uncertainty-aware classification, but their reliance on noise-injected clinical datasets limits exposure to real-world motion artifacts, constraining validity.

Conventional deep learning frameworks have also shown limitations. (Abdelgaber et al., 2023). developed a convolutional LSTM-based autoencoder that achieved an RMSE of 0.35 mV and 0.923 ρ on MIMIC II. However, the model required extensive preprocessing pipelines—including peak detection, beat segmentation, augmentation, and beat stitching—creating additional failure points under noise and complicating end-to-end deployment. Similarly, (Guo et al., 2024), utilized a U-Net–BiLSTM architecture with strong local reconstruction metrics (e.g., RMSE 0.077 mV, ρ 0.861), yet the evaluation was limited to short 3-s windows and still depended on R-peak detection, restricting long-duration applicability and generalization under motion.

In contrast, our transformer-based GAN solution overcomes these limitations through a combination of self-supervised pre-training and fine-tuning, enabling robust subject-independent performance even on datasets rich in motion artifacts. Compared to other models such as CycleGAN (Zhu et al., 2017) and LSTM-based architectures, our Transformer-based GAN approach achieved lower RMSE and higher fidelity in synthesizing ECG signals. The leave-one-subject-out (LOSO) validation strategy further validated our model’s robustness, demonstrating improved performance across different datasets and subject-specific variances. Moreover, none of these previous studies have attempted the following:

- To implement self-supervised method to overcome lower generalization capability issue for ECG generation including fine-tuning for smaller datasets.
- To implement the solution on a dataset including high level of motion artifacts.

In our work, we mainly pioneered to address these issues, hence improved the generation performance.

### 9.2 Remarks on results

The integration of the Transformer model into our GAN framework led to superior performance, primarily due to its attention mechanism, which effectively captures long-range dependencies in sequential data. This capability enhanced the model’s ability to focus on relevant signal components, thereby improving the quality of the generated ECG signals. Additionally, our original dataset employed in this study played a crucial role in this improvement. Its substantial size provided the necessary data diversity to learn intricate features and adequately train the model, which is essential for achieving robust signal generation in data-hungry deep learning approaches.

Among the various window lengths tested, 64-s and 96-s segment lengths provided the best balance between model complexity and synthesis accuracy. This window length captured sufficient cardiac cycle information, optimizing the model’s learning capacity without overfitting. Additionally, longer segments would require more complex models, which are harder to train with limited data. Shorter segments are faster to process, allowing for more training epochs and smaller learning rates.

The results indicate that our method accurately captures the physiological relationship between PPG and ECG signals, as evidenced by lower RMSE and higher Pearson correlation coefficients compared to previous studies. Specifically, the use of Transformer-based GAN architecture with incorporating self-supervised learning significantly improved ECG signal reconstruction quality and allowed our model to achieve better generalization and performance. The self-supervised approach facilitated robust feature extraction, improving the synthesis accuracy, particularly in scenarios involving varying segment lengths and motion artifacts.

We deliberately presented both relatively straightforward and challenging scenarios for ECG generation in Figures 6–8. Figures 6, 8 demonstrate the superior performance of ECG signal generation from minimally distorted (low-noise) PPG signals both from Who is Alyx? and MIMIC III datasets. The MIMIC III dataset, collected within an intensive care unit (ICU) setting, where patients typically remain at rest with limited physical or emotional activity, exhibits stable signal morphology and reduced motion artifacts. As a result, ECG generation from PPG on this dataset is inherently less complex, which is reflected in the elevated metric scores observed. In contrast, the Who is Alyx? includes real-world variability, motion an emotional fluctuations, making ECG generation more challenging, thereby demonstrating the robustness and generalizability of our model across diverse conditions.

Wristband-based measurements often introduce substantial motion artifacts due to their usage during daily activities, making them generally more susceptible to noise compared to chest straps, which offer greater positional stability. Despite the heavy distortion caused by motion artifacts in the PPG signals, as shown in Figure 7, our model exhibits remarkable efficacy in generating ECG signals while preserving temporal variations. Notably, key ECG amplitude features such as the R-peaks, P-waves, and T-waves are accurately reconstructed, even when the PPG signal’s peaks and troughs are affected by motion artifacts. Moreover, although motion artifacts obscure true beat-to-beat intervals in PPG signals, leading to irregular or inconsistent heartbeat timings, the model effectively preserves and reflects heart rate variability.

Having demonstrated AF detection as a practical application achieving 89.2% accuracy when trained with generated ECG data and 90.6% accuracy when evaluated on generated test data, we note that these results may vary depending on several factors, including dataset size, demographic characteristics, the presence of motion artifacts, and the choice of machine learning models. We utilized the MIMIC PERform AF dataset; however, larger and more diverse datasets are likely to further improve performance. Similar considerations also apply to the baseline fluctuation analysis, where increased data diversity and scale may yield more robust and generalizable findings. Synthesizing ECG from PPG signals is advantageous for the healthcare sector, particularly for applications in wearable technology and long-term health monitoring. It provides a cost-effective, non-invasive alternative for continuous cardiac monitoring, facilitating early detection of cardiovascular conditions. The widespread availability of PPG sensors in wearable devices underscores the practical utility of our approach in real-world health monitoring scenarios.

### 9.3 Limitations

Training GAN models in a stable manner poses inherent challenges due to issues such as mode collapse and catastrophic forgetting. To address these challenges, we incorporated a gradient penalty term (with Wasserstein loss, see Figure 5) and leveraged self-supervised learning, respectively, which also aimed to enhance the generalization capability of the models. However, integrating a combination of Transformer and LSTM models within the GAN architecture could potentially result in more stable training and might further improve performance outcomes.

Our model struggled in scenarios involving extreme motion artifacts, which introduced noise that the current architecture could not adequately filter. This suggests a need for more advanced noise-handling techniques or the incorporation of additional data modalities. Future iterations of this model could benefit from integrating accelerometer (ACC) data, which might improve performance by providing context on motion-related noise. ACC data could help differentiate between physiological signal variations due to motion and genuine cardiac events, thus enhancing the fidelity of the generated ECG.

Participant demographic factors such as age, gender, ethnicity, and health status significantly influence both PPG and ECG signals and thus affect the model’s generalization capability. For example, differences in skin tone can impact the accuracy of PPG measurements, while age-related changes in heart rate variability (HRV) could alter ECG patterns. Participants with specific cardiac conditions, such as comorbidities or rare cardiac anomalies, and those influenced by geographic and lifestyle factors (e.g., physical activity levels, diet, stress) may exhibit variations in cardiac signals. A lack of diversity in these factors could result in biased models that fail to generalize effectively across broader populations.

Furthermore, variability among wearable devices in terms of sensor quality, resolution, and sampling frequency can affect the fidelity of PPG and derived ECG signals. For instance, lower-resolution sensors may miss subtle waveform details, reducing the quality of model inputs. The placement of wearable devices on different anatomical locations (e.g., wrist, finger, ear) and environmental factors such as motion artifacts, ambient light, and temperature can also introduce noise or alter PPG signal morphology. Lastly, the absence of standardized calibration protocols across devices could result in discrepancies in collected data, further limiting the model’s generalization capability.

## 10 Conclusion

We demonstrated that our Transformer-based ECG generation model showed superior performance by reducing the heart rate error 83.9% and 0.168 mV RMSE on MIMIC III, 72.4% and 0.22 mV RMSE on Who is Alyx? with help of self-supervised learning. In addition to its relevance for the AI community, our proposed solution holds promise for broader applications in the healthcare and wearable technology sectors, particularly in the realm of continuous health monitoring. Through our practical AF detection experiment, we further provided compelling proof-of-concept evidence that the generated ECG signals carry clinically meaningful information. Cardiac activity monitoring is a crucial component of continuous health monitoring systems, which may facilitate the early diagnosis of cardiovascular diseases. This early detection could, in turn, prompt preventative actions that help mitigate serious cardiac conditions. However, as previously mentioned, there lacks a universally applicable solution for everyday continuous ECG monitoring. Our study addresses this deficiency by employing PPG signals, which can be readily obtained from nearly all commercially available wearable devices. We incorporate these signals into our newly developed Transformer-based GAN framework to accurately capture and generate ECG signals, reflecting users’ cardiac information. This integration aims to bridge the gap in current continuous cardiac monitoring technologies. This model is expected to be used in wearable devices as an effective alternative for a low-cost, long-term health or fitness monitoring application.

### 10.1 Future work

Future work will focus on enhancing model robustness, particularly against motion artifacts, by incorporating ACC data. For example, in the Who is Alyx? dataset, ACC data was collected alongside ECG capture from Polar H10 chest strap. Integrating this data which provides insights into chest movements and breathing-induced motion, may improve ECG generation by providing contextual information for artifact mitigation. Beyond heart rate estimation, the proposed model has potential applications in cardiac health monitoring, including arrhythmia detection, cardiovascular disease diagnosis, and conditions such as atrial fibrillation and ischemia. We also plan to extend its use to new domains, such as emotion recognition (e.g., stress, fear), where generated ECG may offer superior performance over PPG-based methods in wearable systems.

## Data Availability

The datasets presented in this study can be found in the GitHub repository at https://github.com/cschell/who-is-alyx.

## Footnotes

1
https://www.who.int/news-room/fact-sheets/detail/cardiovascular-diseases-(cvds)

2
https://github.com/m1237/self-supervised-ppg-to-ecg.
